# Treatment of Severe Preeclampsia With Eclamptic Seizures in Cesarean Delivery With Postoperative Ionized Magnesium Monitoring

**DOI:** 10.7759/cureus.71741

**Published:** 2024-10-17

**Authors:** Mioko Shibata, Shohei Noguchi, Takao Kato, Koki Kaneko, Katsuo Terui

**Affiliations:** 1 Department of Anesthesiology, Saitama Medical Center, Saitama Medical University, Kawagoe, JPN; 2 Department of Obstetric Anesthesiology, Saitama Medical Center, Saitama Medical University, Kawagoe, JPN

**Keywords:** blood gas analysis, eclampsia, ionized magnesium, magnesium sulfate, preeclampsia

## Abstract

Prevention of recurrent eclamptic seizures requires the administration of magnesium sulfate. However, to our knowledge, there are no reports of cases in which the ionized magnesium concentration has been monitored during magnesium sulfate administration to prevent eclampsia. We describe a case in which monitoring of ionized magnesium permitted the use of magnesium sulfate to prevent a third eclamptic seizure. A 31-year-old primigravida with severe preeclampsia and eclampsia underwent emergency cesarean delivery. The patient had a recurrence of seizures intraoperatively. Postoperatively, she was managed with continuous magnesium sulfate infusion under ionized magnesium monitoring at the bedside using a blood gas analyzer in the intensive care unit. This approach might have helped prevent further seizures and complications associated with the administration of magnesium sulfate, such as hypermagnesemia. This case indicates that in the use of magnesium sulfate for patients with preeclampsia, ionized magnesium measurement was used for the rapid determination of magnesium levels.

## Introduction

A loading dose followed by continuous infusion of magnesium sulfate (MgSO_4_) for 24 hours postpartum is a recommended protocol for preventing eclamptic seizures [1]. Magnesium monitoring is essential to establish effective prophylaxis and prevent adverse effects.

The current prophylactic range for total magnesium (tMg) is considered to be 4.8-8.4 mg/dL [1]. However, this range reflects concentrations at which physiological responses to hypermagnesemia become apparent. It is not a preventive range established by an accumulation of strong evidence that specifically demonstrates the prevention of eclamptic seizures. It is crucial to note that excessively high magnesium concentrations can be detrimental, and sporadic cases of hypermagnesemia due to rapid administration of MgSO_4_ have been reported [2,3]. Measurement of the magnesium concentration from a blood sample typically takes at least one hour to obtain results, and depending on the situation, it may take several hours or more. This makes rapid follow-up challenging. By contrast, ionized magnesium (iMg) can be measured in two minutes using a blood gas analyzer. In addition, the concentrations of tMg and iMg are not always correlated [4], necessitating separate iMg monitoring.

The required plasma magnesium concentration to prevent eclamptic seizures remains unclear and subject to ongoing debate [5]. The prophylactic range refers solely to tMg, while that for iMg, which is widely accepted as the biologically active form of magnesium, remains unknown.

We encountered a case of eclampsia following membrane rupture during cesarean delivery, in which subsequent recurrence of eclampsia was prevented by MgSO_4_ administration under iMg monitoring via blood gas analysis.

## Case presentation

A 31-year-old woman, gravida 1, para 0, with no significant family or medical history was diagnosed with severe preeclampsia and eclampsia at 38 weeks and six days gestation after presenting with high blood pressure (170/120 mmHg) and tonic-clonic seizures at another hospital. She was treated with continuous intravenous infusion of MgSO_4_ at 1 g/h and diazepam 2.5 mg before transport to our hospital. A loading dose of MgSO_4_ was not given at the other hospital due to the inability to monitor blood Mg levels.

Upon arrival at our hospital, her seizures had subsided and her consciousness level was GCS E4V5M6, with a blood pressure of 166/112 mmHg. Blood tests indicated hemoconcentration, elevated liver enzymes, and normal renal function (Table 1).

**Table 1:** Blood tests

	Test value	Reference ranges (Non-pregnant state)
White blood cell count	20.9 ×10^3^ /µL	3.3-8.6 ×10^3^ /µL
Hemoglobin	14.2 g/dL	11.6-14.8 g/dL
Hematocrit	41.50%	35.1-44.4%
Platelet count	156 ×10^3^ /µL	158-348 ×10^3^ /µL
Albumin	3.1 g/dL	4.1-5.1 g/dL
Sodium	132 mEq/L	138-145 mEq/L
Potassium	3.9 mEq/L	3.6-4.8 mEq/L
Chloride	102 mEq/L	101-108 mEq/L
Total magnesium	2.1 mg/dL	1.8-2.4 mg/dL
Aspartate transaminase	137 U/L	13-30 U/L
Alanine transaminase	109 U/L	7-23 U/L
Lactate dehydrogenase	369 U/L	124-222 U/L
Total bilirubin	0.8 mg/dL	0.4-1.5 mg/dL
Creatinine	0.61 mg/dL	0.46-0.79 mg/dL
Urea nitrogen	9 mmol/L	8-20 mmol/L

**Figure 1:**
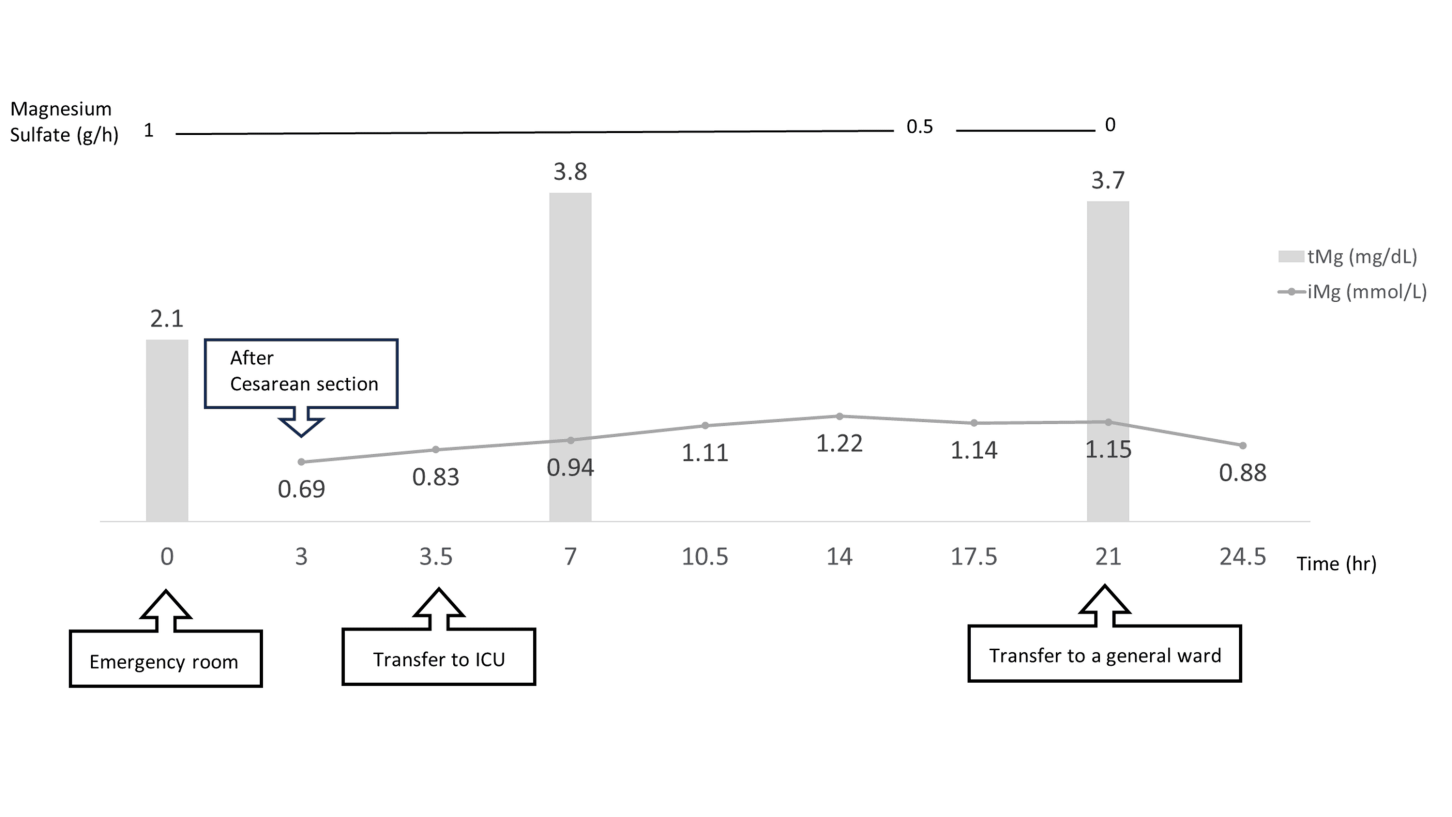
Trends in perinatal serum magnesium concentrations in this case tMg (mg/dL): total serum magnesium concentration (reference range, 1.8-2.5 mg/dL); iMg (mmol/L); ionized magnesium concentration (reference range, 0.45-0.67 mmol/L); ICU: intensive care unit

MgSO_4_ infusion was continued at 1 g/h. After excluding intracranial lesions using brain computed tomography, an emergency Cesarean delivery was planned due to maternal eclampsia. The cesarean delivery was performed under spinal anesthesia with 12 mg of hyperbaric bupivacaine, 0.15 mg of morphine, and 10 µg of fentanyl at the L3/4 intervertebral space. The operative time was 1 hour 24 minutes, with blood loss of 413 g and urine output of 350 mL. Forty minutes after childbirth, the patient developed tonic-clonic seizures, upward deviation of the eyes, and apnea. Oxygen was administered and the airway was secured with manual ventilation support of the oral airway and intravenous diazepam 2.5 mg. Recurrent seizures occurred after three hours after the first seizures and ceased within about one minute, with no recurrence thereafter. In the intensive care unit (ICU), MgSO_4_ administration was continued with reference to iMg levels measured with a blood gas analyzer (STAT PROFILE pHOx UltraTM, Nova Biomedical, Waltham, MA, USA) every 3.5 hours. MgSO_4_ was administered at a rate of 1 g/h, and after 17 hours, the dose was reduced to 0.5 g/h in order to keep the iMg level below 1.34 mmol/L, which is twice the upper limit of the iMg reference range. MgSO_4_ was administered for up to 24 hours after surgery (Figure 1).

At two points during the course, where iMg and tMg were measured simultaneously, the iMg/tMg ratios were 0.59 and 0.75, respectively. In the ICU, the patient was sedated with dexmedetomidine, with her consciousness level on the Richmond Agitation-Sedation Scale maintained between -1 and 0 and pain control of 0 on a numerical rating scale. She was transferred to the general ward on postoperative day (POD) 1. Brain magnetic resonance imaging on POD 1 showed no evidence of transient posterior reversible encephalopathy syndrome or reversible cerebral vasoconstriction syndrome. Blood pressure decreased to within the reference range during the cesarean section and was well controlled until POD 2. On POD 3, hypertension developed with a systolic blood pressure of 150 mmHg, and an oral calcium channel blocker (nifedipine, 20 mg two times daily) was started. The patient was discharged on POD 6. The neonatal Apgar scores were 8 at one minute and five minutes, and the umbilical cord arterial pH was 7.203 at birth. The neonate required intubation and neonatal ICU admission due to insufficient respiratory effort. On day 1, respiratory effort improved, the tracheal tube was removed, and the neonate was managed on a high-flow nasal cannula with FiO_2_ of about 0.3. Oxygen support was slowly withdrawn and the neonate was discharged to home on day 16. 

## Discussion

We experienced a case of eclampsia that occurred after membrane rupture during a cesarean delivery, where the recurrence of eclampsia was successfully prevented by administering MgSO_4_ while monitoring iMg levels through blood gas analysis.

The average iMg/tMg ratio in healthy individuals is 0.64 [6,7]. However, the tMg and iMg concentrations do not always correlate [4], which may be due to variations in total protein concentration, pH [7,8], patient factors, and certain diseases [9]. A poor correlation between iMg and tMg has been found in pregnant women with severe preeclampsia and eclampsia undergoing MgSO_4_ therapy [10]. Although there are some reports of a correlation between iMg and tMg in pregnant women with eclampsia or preterm labor [11], the evidence is not sufficient to predict iMg based solely on the measurement of tMg. The iMg/tMg ratio ranged from 0.59 to 0.75 in this case, despite the measured tMg being almost identical (3.8 and 3.7 mg/dL), which suggests that predicting iMg from tMg is challenging. It is also noteworthy that pregnant women can have 20% lower iMg levels than non-pregnant women at the same tMg concentration [12]. Moreover, an iMg/tMg ratio of less than 0.25 may indicate a risk of preeclampsia [13]. It is still unclear why iMg values differ between pregnant and non-pregnant women, but monitoring of iMg rather than tMg may be more appropriate for diagnosis and treatment of preeclampsia with MgSO_4_.

Blood gas analysis is helpful for convenient and rapid monitoring of iMg. Biochemical blood tests cannot measure tMg levels quickly, and there have been sporadic reports of cases of hypermagnesemia due to rapid MgSO_4_ administration [2,3]. This may partly explain why a loading dose of MgSO_4_ was not administered at the time of the initial seizure (at another hospital) in this case. Thus, we suggest that monitoring of iMg is important because it is the physiologically active form, and knowledge of the iMg level is required for timely follow-up. This case indicates that the iMg concentration can be monitored in a timely manner. The iMg reference range is 0.45 to 0.67 mmol/L, and iMg has been found to be safe up to twice the upper limit [14,15]. In simultaneous measurements of tMg and iMg in 50 pregnant women with preeclampsia treated with MgSO_4_, most patients maintained levels of 2-6 mg/dL for tMg and 0.5-1.5 mmol/L for iMg [11]. These data differ slightly from the tMg level of 4.8-8.4 mg/dL, which is considered to be the target range for tMg in the treatment of preeclampsia. In the ICU, MgSO_4_ was administered at 1 g/h, aiming for an iMg level below 1.34 mmol/L, which is twice the upper limit of the standard iMg. When this value was predicted to be exceeded, the dose was reduced to 0.5 g/h. As a limitation, the conversion ratio between iMg and tMg is not fixed and may vary depending on the stage of pregnancy or the severity of preeclampsia. Consequently, further evidence is necessary to accurately determine the iMg concentration required to prevent eclamptic seizures. Accumulation of data on iMg concentrations during eclampsia treatment may identify a clear therapeutic range for preventing eclamptic seizures and could also help to define treatment ranges for impending premature birth. One of the drawbacks of measuring iMg is that the monitoring device is not widely used. However, as with the history of ionized calcium, this issue will be solved as more clinicians recognize the importance and usefulness of iMg monitoring.

## Conclusions

We reported a case of eclamptic seizures associated with severe preeclampsia. While the administration of MgSO₄ under iMg monitoring using a blood gas analyzer allowed for rapid and precise control of blood iMg levels, potentially aiding in the prevention of recurrent seizures, this observation is descriptive. Further accumulation of data on iMg concentrations during eclamptic prevention will be necessary to determine a clear effective range for iMg.
